# Altered Thyroid Function Tests Observed in Hypophosphatasia Patients Treated with Asfotase Alfa

**DOI:** 10.1155/2021/5492267

**Published:** 2021-10-28

**Authors:** Hajime Kato, Naoko Hidaka, Minae Koga, Yuka Kinoshita, Masaomi Nangaku, Noriko Makita, Nobuaki Ito

**Affiliations:** ^1^Division of Nephrology and Endocrinology, The University of Tokyo Hospital, 7-3-1 Hongo, Bunkyo-ku, Tokyo 113-8655, Japan; ^2^Osteoporosis Center, The University of Tokyo Hospital, 7-3-1 Hongo, Bunkyo-ku, Tokyo 113-8655, Japan

## Abstract

**Background:**

Asfotase alfa is the only approved treatment that can normalize mineralization in patients with hypophosphatasia (HPP). Its interference in alkaline phosphatase (ALP) dependent immunoassays has been reported.

**Objective:**

To describe thyroid function tests interfered with by asfotase alfa and elucidate the underlying mechanism. *Patients and Methods*. Three patients with HPP treated with asfotase alfa were included. Thyroid hormone levels measured using five different immunoassays with or without ALP as a labeling enzyme during asfotase alfa treatment were evaluated.

**Results:**

After the initiation of asfotase alfa, three HPP patients showed low free triiodothyronine (FT3) and free thyroxine (FT4) measured with AIA-2000 (Tosoh, Tokyo, Japan), an enzyme immunoassay system that uses ALP as a labeling enzyme, but their thyroid-stimulating hormone (TSH) levels were within the normal range. The other CLEIA system using ALP as a label, AIA-CL2400 (Tosoh, Tokyo, Japan), and ALP-independent immunoassay systems demonstrated normal FT3 and FT4 levels. These data suggested that although the thyroid function of these three patients was normal, asfotase alfa interfered with the thyroid hormone measurements made with AIA-2000. AIA-2000 and AIA-CL2400 adopted one-step and delayed one-step measurements, respectively, and the same antibody was used for both immunoassays. However, asfotase alfa may be absorbed on the magnetic beads used in the AIA reagent with the AIA-2000 system but not absorbed on the microparticles used in AIA-CL2400.

**Conclusion:**

Clinicians should be aware of the possible interference in thyroid function measurements by adopting specific types of immunoassays in asfotase alfa-treated HPP patients.

## 1. Introduction

Hypophosphatasia (HPP) is an inherited disorder caused by loss-of-function mutations of the gene that encodes tissue-nonspecific alkaline phosphatase (TNSALP), which is also known as ALPL [1–3]. Mutations in ALPL are transmitted in an autosomal dominant or recessive manner and result in reduced enzymatic activity of TNSALP, which leads to the accumulation of its substrates, including inorganic pyrophosphate (PP_i_) [3–5]. Because extracellular accumulation of PP_i_ prevents mineralization or leads to deposition of pyrophosphate crystals in the joints, patients with HPP exhibit a wide spectrum of clinical symptoms, including rickets/osteomalacia and calcium pyrophosphate deposition disease [6, 7]. HPP is categorized into seven forms based on the onset and severity of the symptoms: odonto, adult, mild childhood, severe childhood, infantile, perinatal, and benign perinatal HPP [8]. Among Japanese people, the most prevalent ALPL mutation was c.1559delT, and the c.1559delT carrier frequency was 1/480 [9]. The homozygous ALPL mutation c.1559delT was associated with perinatal HPP, and the compound heterozygous *ALPL* mutation F310L was related to benign prenatal HPP in Japan [10].

Asfotase alfa (Strensiq®, Alexion Pharmaceuticals, Inc.) consists of alkaline phosphatase (ALP) and Fc regions of the antibody conjugated with deca-aspartate, and it is the only treatment method available that can improve impaired mineralization in the bone among patients with HPP and has become available in many countries, including Japan, Canada, the United States, and the European Union [11].

ALP is also commonly used as a label substance in enzyme-linked immunosorbent assays (ELISAs) for routine measurements of hormones and other biomarkers. In immunoassays that use ALP for signal amplification, ALP hydrolyzes substrates (paranitrophenylphosphate (pNPP), 4-methylumbelliferyl phosphate phosphatase (4-MUP), nitro blue tetrazolium salt and 5-bromo-4-chloro-3-indolyl-phosphate (NBT/BCIP), or nicotinamide adenine dinucleotide phosphate (NADPH)) and creates a color/fluorescence change that is measured with a spectrophotometer. In the sandwich ELISA system, the amount of enzyme is directly proportional to the amount of measured biomarkers. On the other hand, in the competitive ELISA, the quantity of antigen in the sample is inversely correlated with the amount of enzymatic activity.

Because asfotase alfa contains an ectodomain region of ALP that is similar to the ALP used in some ELISAs, asfotase alfa has been reported to have the potential to interfere with ELISAs using ALP-labeled tracers. To date, a few case reports have shown that asfotase alfa may interfere with several laboratory tests, such as testosterone, cardiac troponin I, *β*-human chorionic gonadotropin, and oxytocin [12–14]. In this report, three cases of adults with HPP under treatment with asfotase alfa were introduced, whose thyroid hormone measurements were obviously affected by asfotase alfa depending on the type of assay system.

## 2. Patients and Methods

### 2.1. Patients

Three patients with HPP who regularly visited our institute were included in the present study. All procedures were performed in accordance with the ethical standards of the Declaration of Helsinki and approved by the institutional ethical board of the University of Tokyo Hospital (refs. 2879 and 11221), and all participants provided written informed consent.

### 2.2. Patient Data Collection

Patient data including age, diagnosis, the result of the genetic analysis, timing of the initiation of asfotase alfa, and thyroid functions were extracted retrospectively.

### 2.3. Measurement of FT3, FT4, and TSH Levels Using Various Immunoassay Systems

Thyroid-stimulating hormone (TSH), free triiodothyronine (FT3), and free thyroxine (FT4) in the three adult participants with HPP with or without asfotase alfa were measured in five immunoassay systems: AIA-2000 (Tosoh, Tokyo, Japan), AIA-CL2400 (Tosoh, Tokyo, Japan), AccuraSeed (FUJIFILM Wako Pure Chemical Corporation, Osaka, Japan), ARCHITECT i2000SR (Abbott Diagnostics, Chicago, Illinois), and Cobas 8000 e801 (Roche Diagnostics, Rotkruez, Switzerland) (Table 1). AIA-2000 and AIA-CL2400 were based on one-step and delayed one-step measurements, respectively, and both immunoassays utilized ALP as a labeled substance. AccuraSeed and ARCHITECT i2000SR measure thyroid hormones using a two-step method and utilize peroxidase and acridinium ester as signals, respectively. Thyroid hormone measurement with Cobas 8000 e801 adopts a one-step measurement method and utilizes ruthenium as a substrate. Although both AIA-2000 and AIA-CL2400 adopt the same antibody for immobilization and signaling, AIA-CL2400 was modified from AIA-2000 as follows. The AIA-CL2400 measurement method was changed from enzyme immunoassay (EIA) to chemiluminescent enzyme immunoassay (CLEIA). In addition, for FT3 and FT4 measurements, a delayed one-step assay was performed on AIA-CL2400 instead of a one-step assay on AIA-2000. In addition, magnetic beads, which are used for immobilization of the primary antibody in the AIA-2000 system, were changed to magnetic microparticles in the AIA-CL2400 system.

## 3. Results

### 3.1. Case Reports

**Case 1 .**

The first case was a 67-year-old woman who presented with periarticular calcification and was diagnosed with adult-onset HPP with iliac crest biopsy that demonstrated impaired mineralization. Mutational analysis showed a heterozygous pathogenic single nucleotide deletion in ALPL, c.1559delT (p.Leu520ArgfsX86). Replacement therapy with asfotase alfa (240 mg/week: 4 mg/kg/week) has been administered since the patient was 65 years old.

**Case 2 .**

Case 2 was a 23-year-old man with infantile severe HPP whose genetic test revealed a compound heterozygous mutation in ALPL, c.670A > G (p.Lys224Glu) and c.1276G > T (p.Gly426Cys). He has been treated with asfotase alfa (240 mg/week: 6 mg/kg/week) since 17 years of age.

**Case 3 .**

The patient was a 22-year-old woman with benign prenatal HPP. Genetic testing revealed that she had compound heterozygous mutations in ALPL, c. 979T > C (p. Phe310Leu) and c.1559delT (p.Leu520Argfs86). Treatment with asfotase alfa (240 mg/week: 6 mg/kg/week) was initiated at the age of 19 years.

### 3.2. Thyroid Function before and after Asfotase Alfa Treatment

Because all three cases complained of fatigue, which is a symptom of HPP with or without asfotase alfa treatment, thyroid function (TSH, FT3, and FT4) was evaluated at the laboratory at the University of Tokyo Hospital. Of all three cases, normal thyroid functions were confirmed with an ALP-mediated measuring/signaling system (AIA-2000) before the initiation of asfotase alfa in cases 1 and 3, and there was no record of thyroid function for case 2 before treatment. However, thyroid function measurements using the AIA-2000 system after the initiation of asfotase alfa (case 1: 17 months; case 2: 6 years; case 3: 3 years) revealed abnormally low FT3 and FT4 levels with concomitant inappropriately normal TSH levels without any typical symptoms of hypothyroidism (Table 2). The immunoassay system for measuring TSH, FT3, and FT4 changed in our institute during the study period, and thyroid function was measured using the new immunoassay system (AIA-CL2400), which also adopted ALP as a signal. These measurements revealed TSH, FT3, and FT4 levels within the normal range (Table 2). The obvious difference in thyroid hormone levels between the two analyzers, both of which adopted ALP for signal enhancement and identical antibody sets, prompted us to measure FT3 and FT4 levels in these three HPP patients undergoing asfotase alfa treatment with other analyzers utilizing different measuring systems, including AccuraSeed, ARCHITECT i2000SR, and Cobas8000 e801 **(**Table 1**)**. All immunoassay systems other than AIA-2000 demonstrated normal TSH, FT3, and FT4 levels, which indicated normal thyroid function in the three participants with HPP undergoing asfotase alfa treatment and interference between asfotase alfa and AIA-2000 in an antibody-independent manner (Table 2).

## 4. Discussion

In the present report, a thyroid function test using AIA-2000, an immunoassay system that uses ALP as a labeling enzyme, revealed abnormally low levels of FT3 and FT4 despite normal TSH levels in three patients with HPP treated with asfotase alfa. On the other hand, a thyroid function test using AIA-CL2400, another immunoassay system using the same antibodies and ALP as a label, and an ALP-independent immunoassay analyzer revealed normal FT3 and FT4 levels. Asfotase alfa may be absorbed on the magnetic beads used in the AIA-2000 system, which explains the interference observed exclusively in thyroid function tests performed with AIA-2000 but not AIA-CL2400.

**Figure 1:**
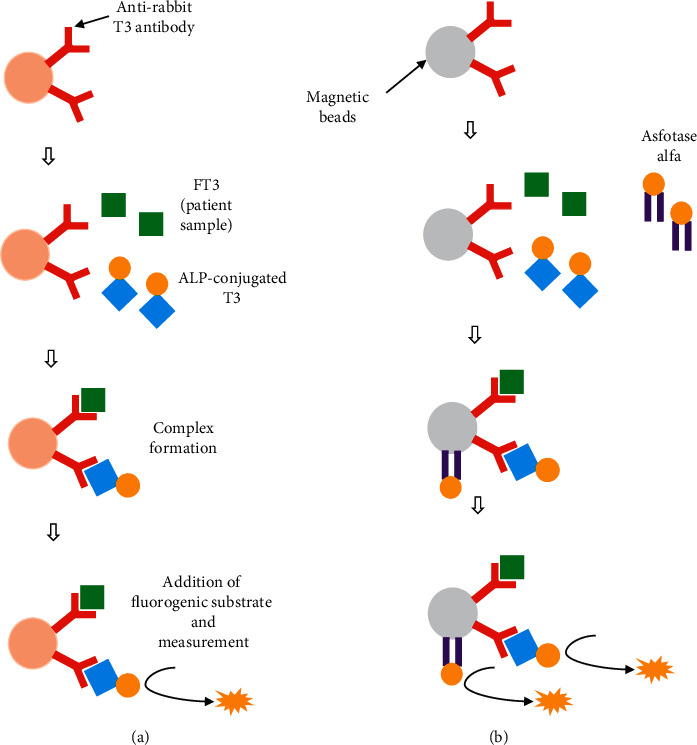
Schematic images of how asfotase alfa interferes with the measurement of thyroid hormone (FT3) levels in AIA-2000. (a) Normal methods used to assess FT3 levels, including AIA-CL2400. (b) Possible interference mechanism in the AIA-2000 system. Asfotase alfa inappropriately binds to magnetic beads, leading to spurious thyroid function results.

Occasionally, different substances interfered with the results of some immunoassay systems by various mechanisms, such as analyte-independent interference or analyte-dependent interference [13, 15]. Analyte-independent interference is typically caused by lipemia, whereas analyte-dependent interference is attributed to various causes, including heterophilic antibodies, human antianimal antibodies, complement, lysozyme, and paraprotein [15]. Whether the interfering substances result in falsely low or falsely high values depends on both the types of immunoassay systems (noncompetitive or competitive) and the similarity of the structures between the measured substance and the interfering substance [16]. In the present assay system, AIA-2000, which reported falsely low thyroid hormone levels, the interaction between ALP conjugated with T3 or T4 and 4-MUP was utilized as a signaling system. Because the principle of the FT3 assay involves a competitive enzyme immunoassay (EIA), ALP conjugated with thyroid hormones, which remained after incubation and washing, was inversely proportional to the thyroid hormone levels in the serum sample (Figure 1(a)). When asfotase alfa was not completely removed from the sample after incubation and washing cycles, the remaining exogenous ectodomain of ALP as a part of asfotase alfa resulted in falsely low FT3 and FT4 levels.

Two mechanisms that explain the residual asfotase alfa after washing have been reported. The first mechanism was the inadequate washing of unbound analyte. Laboratory tests of thyroid hormones are well known to be affected by exogenous autoantibodies or medications [17]. Additionally, previous reports showed that endogenous elevated ALP levels resulted in spuriously high cardiac troponin I and *β*-human chorionic gonadotropin when measured with an assay system that used ALP as a substrate [12]. The second mechanism involves the inappropriate interaction between asfotase alfa and the primary antibody of the assay. Asfotase alfa contains the Fc region of human immunoglobulin G, which is the catalytic domain of ALP. When the Fc region of asfotase alfa and any antibody used in the assay shares a similar structure, the interaction between asfotase alfa and the antibody results in a false measurement value. A previous study reported 62% similarity in the amino acid sequence between the Fc regions of asfotase alfa and mouse antitestosterone antibody, the primary antibody that is recognized by the goat anti-mouse polyclonal antibody coating the paramagnetic particles utilized in the assay [13].

Although the two abovementioned mechanisms have been reported, both mechanisms failed to explain the discrepancy in thyroid hormone levels between the two analyzers that utilized ALP for signal enhancement. AIA-2000 and AIA-CL2400 adopted one-step and delayed one-step measurements, respectively, and both measurement methods had similar washing protocols. Additionally, both analyzers used the same antibody set. Given that FT3 and FT4 measurements obtained with the AIA-2000 and AIA-CL2400 systems utilized magnetic beads with primary antibody and magnetic microparticles with primary antibody, respectively, this difference could explain the discrepancy in FT3/FT4 measurements (Figure 1(b)). In fact, the manufacturer confirmed by *in vitro* assays that the magnetic beads from AIA-2000 exhibited a high affinity for asfotase alfa, whereas the magnetic microparticles used in AIA-CL2400 did not have any affinity for asfotase alfa. No interference between ALP and magnetic beads or between ALP and magnetic microparticles was observed, suggesting specific interference between asfotase alfa and magnetic beads used in AIA-2000 (data not shown). However, the detailed study protocol and actual measurement values cannot be disclosed under a confidentiality agreement.

Although this study revealed the direct absorption of asfotase alfa to magnetic beads as a novel potential mechanism explaining the altered thyroid hormone levels observed in specific types of ALP-based immunoassay systems, there were some limitations. First, detailed measurement methods, including precise structures of magnetic beads in AIA-2000 and magnetic microparticles in AIA-CL2400, were not fully disclosed due to the manufacturer's regulations, and the details of the experimental results comparing the affinities between asfotase alfa and magnetic beads and magnetic microparticles performed by the manufacturer could not be disclosed in this study. Second, only two of the ALP-based analyzers were included in the present study, and the same interfering mechanism described in the present study was not validated in any of the other ALP-based analyzers. Further studies are therefore warranted to analyze asfotase alfa interference in thyroid function tests using other ALP-based assay systems.

The present study revealed that asfotase alfa could affect some of the ALP-based immunoassays using specific types of magnetic beads that have an affinity for asfotase alfa. Particularly, this was the first report describing the novel interference between asfotase alfa and the thyroid function test. Clinicians should be aware of the possible interference in thyroid function measurements by adopting specific types of magnetic beads in asfotase alfa-treated HPP patients.

## Figures and Tables

**Table 1:** The characteristics of the immunoassay systems tested in the present cases.

Analyzer	Reference range			Assay principle		
	TSH (*μ*IU/mL)	FT3 (pg/mL)	FT4 (ng/mL)	TSH	FT3, FT4	Detection
AIA-2000	0.38–4.31	2.1–3.8	0.82–1.63	Sandwich EIA	Competitive EIA	ALP/4-MUP
AIA-CL2400	0.61–4.23	1.72–3.44	0.71–1.69	Sandwich CLEIA	Competitive CLEIA	ALP/DIFURATR
Accuraseed	0.50–4.80	2.51–4.16	0.88–1.74	Sandwich CLEIA	Competitive CLEIA	Peroxidase
ARCHITECT i2000SR	0.35–4.94	1.68–3.67	0.70–1.48	Sandwich CLIA	Competitive CLIA	Acridinium ester
Cobas 8000 e801	0.50–5.00	2.30–4.00	0.90–1.70	Sandwich ECLIA	Competitive ECLIA	Ruthenium

TSH: thyroid hormone stimulating hormone, FT3: free triiodothyronine, FT4: free thyroxine, CLIA: chemiluminescent immunoassay, CLEIA: chemiluminescent enzyme immunoassay, EIA: enzyme immunoassay, ECLIA: electrochemiluminescence immunoassay, ALP: alkaline phosphatase, 4-MUP: 4-methylumbelliferyl phosphate.

**Table 2:** Thyroid function in three cases assayed by different analyzers before and after asfotase alfa treatment.

	Thyroid function after asfotase alfa		
Case 1^*a*^	TSH (*μ*IU/mL)/%ULN	FT3 (pg/mL)/%ULN	FT4 (ng/mL)/%ULN
AIA-2000	4.24/98	**1.30** ^*c*^/34	**0.22** ^*c*^/13
AIA-CL2400	2.88/12	2.30/67	1.73^*d*^/102
Accuraseed	—	3.28/79	1.53/88
ARCHITECT	1.45/29	2.39/65	1.45/98
Cobas	2.99/60	2.44/61	1.08/64
Case 2	TSH (*μ*IU/mL)/%ULN	FT3 (pg/mL)/%ULN	FT4 (ng/mL)/%ULN
AIA-2000	1.54/36	**0.50** ^*c*^/13	**0.10** ^*c*^/6
AIA-CL2400	1.49/6	2.90/84	0.89/53
Accuraseed	1.60/33	3.59/86	0.88/51
ARCHITECT	0.82/17	3.20/87	0.85/57
Cobas	1.54/31	3.44/86	0.97/57
Case 3^*b*^	TSH (*μ*IU/mL)/%ULN	FT3 (pg/mL)/%ULN	FT4 (ng/mL)/%ULN
AIA-2000	3.03/70	**1.80** ^*c*^/47	**0.19** ^*c*^/11
AIA-CL2400	1.70/7	2.20/64	1.08/64
Accuraseed	1.10/23	3.44/83	1.25/72
ARCHITECT	0.81/16	2.40/65	0.93/63
Cobas	1.48/30	2.44/61	1.14/67

TSH: Thyroid hormone stimulating hormone, FT3: free triiodothyronine, FT4: free thyroxine, %ULN: % upper limit of the normal range, ARCHITECT: ARCHITECT i2000SR, Cobas: Cobas 8000 e801, ^*a*^Thyroid function measured by AIA-2000 before asfotase alfa yielded results within the normal range (TSH: 2.52 *μ*IU/mL, FT3: 2.30 pg/mL, FT4: 1.23 ng/mL)^*, b*^Thyroid function measured by AIA-2000 before asfotase alfa yielded results within the normal range (TSH: 1.32 *μ*IU/mL, FT3: 2.60 pg/mL, FT4: 1.14 ng/mL), ^*c*^Below the reference range, ^*d*^Above the reference range.

## Data Availability

The data supporting the findings of this study are available from the corresponding author upon reasonable request.
